# Aggregate population-level models informed by genetics predict more suitable habitat than traditional species-level model across the range of a widespread riparian tree

**DOI:** 10.1371/journal.pone.0274892

**Published:** 2022-09-19

**Authors:** Shannon L. J. Bayliss, Monica Papeş, Jennifer A. Schweitzer, Joseph K. Bailey

**Affiliations:** Department of Ecology & Evolutionary Biology, University of Tennessee, Knoxville, TN, United States of America; University of Molise, Isernia, ITALY

## Abstract

Identifying and predicting how species ranges will shift in response to climate change is paramount for conservation and restoration. Ecological niche models are the most common method used to estimate potential distributions of species; however, they traditionally omit knowledge of intraspecific variation that can allow populations to respond uniquely to change. Here, we aim to test how population X environment relationships influence predicted suitable geographic distributions by comparing aggregated population-level models with species-level model predictions of suitable habitat within population ranges and across the species’ range. We also test the effect of two variable selection methods on these predictions–both addressing the possibility of local adaptation: Models were built with (a) a common set, and number, of predictors and, (b) a unique combination and number of predictors specific to each group’s training extent. Our study addresses the overarching hypothesis that populations have unique environmental niches, and specifically that (1) species-level models predict more suitable habitat within the ranges of genetic populations than individual models built from those groups, particularly when compared models are built with the same set of environmental predictors; and (2) aggregated genetic population models predict more suitable habitat across the species’ range than the species-level model, an = d this difference will increase when models are trained with individualized predictors. We found the species models predicted more habitat within population ranges for two of three genetic groups regardless of variable selection, and that aggregated population models predicted more habitat than species’ models, but that individualized predictors increased this difference. Our study emphasizes the extent to which changes to model predictions depend on the inclusion of genetic information and on the type and selection of predictors. Results from these modeling decisions can have broad implications for predicting population-level ecological and evolutionary responses to climate change.

## Introduction

Distribution models are a valuable tool for predicting species range dynamics in response to environmental changes, but an overwhelming number of ecological niche models (ENMs) ignore intraspecific variation [1–3]. This is highly problematic given the rate of climate change, advances in eco-evolutionary theory, and the frequency of distribution model use in the literature [4, 5]. Consequently, models disregard the potential for key genotype-by-environment (G x E) and biotic (G x G) interactions across landscapes (G x G x E) [6–8]. Researchers can incorporate intraspecific variation into distribution models with knowledge of phenotypic groups, taxonomic units, genetic groups, or biogeographic regions [2, 9] and intraspecific models can then be compared to each other and to species-wide models for a better understanding of lineage-level differences in climatic-drivers of distribution and potential consequences of climate change [2, 10]. With information from these types of model comparisons, however, we can better identify where, and which, populations, communities, and ecosystems are most at risk to climate change [11–13].

One way to consider intraspecific variation in spatial models is to incorporate underlying genetic sub-structure when delineating the geographic areas to represent the range extent in the models. Species are *not* uniform entities across their geographic distributions, with common examples of locally adapted populations spread across geographic ranges and environmental gradients [14–16]. Accommodating this information in models is critical as locally adapted populations are likely to respond differently to environmental change, which would have implications for shifting species’ distributions and range limits [10, 12, 13, 17–19]. Differing responses of separate populations to environmental change thus violates the major assumption of traditional ENMs that the niche of a species is also the niche of distinct populations [20]. Evidence to date suggests that including this information in ENMs can produce better performing models [21–24], improved transferability of models [25], and broader [13, 19, 26] predictions of distributions in future climate scenarios [1, 2, 24, 27, 28]. On the other hand, splitting occurrence data can often result in datasets that are too small to perform well, suggesting this approach may be best taken only when researchers have *a priori* knowledge of niche divergence or local adaptation [3, 29] and especially with knowledge of the genomic basis of local adaptation [9]. Incorporating genetic structure into spatial models can help address limitations of correlative models relating abiotic variables to species occurrences [27, 30, 31]. For instance, these traditional methods may perform well at predicting current distributions [32] but provide little insight into the mechanisms underlying how and why species are distributed across environments and how future distributions may change [27, 33, 34].

The choice of relevant environmental predictors is important for the utility of models, especially as genetically distinct populations may be locally adapted or have varying tolerances to environmental stressors [2]. Assessments of environmental predictor choice in ENMs consistently show differences in model performance or transferability to future climatic conditions or different geographic regions. Predictor choice (e.g., bioclimatic variables vs. land-use variables) affects model performance and thereby confidence in future predictions [35]. Model performance often improves with the inclusion of species-relevant predictors [36], species ecological traits [37], or ecosystem functioning variables (“EFAs”) [38–40]. Nevertheless, most models assume climate is the main driver of species distributions at a large scale and rely solely on bioclimatic variables available from the Worldclim project [41, 42]. Though these variables can typically describe a species’ current range [43], they may limit the accuracy of predictions across space or time, whereas species-specific predictor variables may improve these predictions [34, 40, 42, 43]. Though assumptions about local adaptation to environmental conditions cannot be drawn simply by incorporating genetic structure into spatial models and examining important variables [2, 44, 45], it is still important to consider that different environmental variables are likely to be relevant for genetically differentiated populations [46]. Variable selection is thus of utmost importance to predictions and conclusion made from these models [47].

Consistent with pervasive G x E interactions known to exist on the landscape [8, 48], this study aims to test the degree of uncertainty in geographic predictions that may arise from manipulating factors related to species’ genetic variation and environmental predictor variables in ENMs. Using known occurrences of genetically differentiated *Populus angustifolia* populations across the species distribution and Maxent modeling algorithms [30], we compare geographic and environmental niche overlap of ENMs. We manipulated two factors in models: (1) *the geographic training extent* using knowledge of *P*. *angustifolia* genetic substructure, and (2) *variable selection method*, providing models with either a unique set of variables selected within the respective population’s geographic training extent, or a common set of variables selected across all training extents (the species range). We expect this selection process will matter greatly when making projections outside of a population’s geographic range. For example, uniquely selecting predictor variables within a population extent may allow for the inclusion of variables in population models that may have been omitted if selected at the species-level.

We test the overarching hypothesis that populations have unique environmental niches, and with our model manipulations, we make two hypotheses. First, (H1) we hypothesize that traditional species models would predict more suitable habitat within the ranges of genetic populations than individual models built from those groups, particularly when compared models are built with the same set of environmental predictors. We expect this because a species model is built on a range of environmental conditions that encapsulates tolerances of all populations. As a corollary, (H2) we hypothesize that aggregated genetic population models would predict more suitable habitat across the species’ range than the traditional species’ model, and that this difference would increase when individual population models were built with unique sets of environmental predictors. We expect this because population models built with unique environmental predictors may capture locally adapted population tolerances to more extreme environmental variables on the edges of species ranges, thus widening potential suitable habitat when aggregated with other population models and projected across the entire species range.

## Materials and methods

### Species occurrence data

The dominant riparian tree, *P*. *angustifolia* James, is a model system for incorporating intraspecific variation into ENMs: the species spans broad abiotic gradients and approximately 1700 km of latitude in the Western United States, across which at least three genetic populations exist [49–52]. As *P*. *angustifolia* is a riparian tree, it is important to include non-climatic hydrological variables that are known to affect the evolution, ecology, and distribution of this and other riparian species [53–56]. Further, the species has strong effects on community interactions and ecosystem functions across its geographic range [8, 57], making it important to understand the extent to which predictions differ with environment and genetic structure. The occurrence dataset for *P*. *angustifolia* was collected May—June 2012 and in June 2021. Latitude and longitude coordinates were collected for each sampled tree as decimal values using Oregon 500 Garmin GPS units with WGS 84 datum. Details of sampling from 2012 have been published previously [58] but, in summary, occurred along elevational gradients of over 17 rivers in the Western United States to span the range of three genetic populations, as determined previously by simple sequence repeat SSR loci [49]. These populations are differentiated around geographic features including the Great Basin, the Rocky Mountains, and the Mogollon Rim [49, 50]. The sampling methods were designed to cover a range of broad environments as well as many locations near the edge of the species’ geographical range. In contrast to random sampling across a species range, these methods are thought to provide more resolved predictions of range expansions and contractions expected with climate change [17, 27, 59, 60].

Before correcting for sample selection bias, the occurrence dataset included georeferenced locations from 725 individual trees. Because *Populus angustifolia* is confined to narrow riparian habitats, 625 of these records were removed so there would be no pseudo-replication within cells of environmental data at 4 km resolution (described below). This left 100 records from which to build our models.

### Environmental variable sources

We used environmental variables from two sources, all verified to be of the same extent (geographic bounds) and spatial resolution (4 km) and projected in the same coordinate system as the species occurrence data (WGS84). First, we extracted bioclimatic variables from 1961–1990 from the AdaptWest Project [v.7.21; 61] which uses data from PRISM and WorldClim to develop informational resources to plan for climate adaptation in North America [61, 62]. Second, we extracted hydrologic variables from the National Hydrography Dataset [NHDPlus; 63, 64], a companion to the Watershed Boundary Dataset that we used to delineate geographic training extents. We used the “near’ function in ArcMap [65] to retrieve values from the nearest stream feature. This second dataset describes river and stream attributes of the riparian habitats of *P*. *angustifolia*. It is important to include these variables as stream properties affect the ecology and evolution, including dispersal ability, of riparian plants like *P*. *angustifolia* [53, 54, 56]. Nine of the available bioclimatic variables were excluded based on knowledge of the species natural history and preliminary model runs (degree days above and below 18 degrees C, and below 0 degrees C; Julian day on which frost-free period begins and ends; extreme 30-year minimum and maximum temperatures; the frost free period; and mean annual solar radiation) and stream velocity was excluded based on a lack of data at some of the nearest stream features.

### Modeling approaches

#### Maximum entropy (Maxent) modeling

Maximum entropy (Maxent) is a high-performing modeling technique that approximates species niches using environmental parameters and performs particularly well with presence-only occurrence data [30, 32]. We ran eight Maxent (Version 3.4.1) computational experiments to address our hypotheses, manipulating the geographic training extent and variable selection method (4 geographic extents x 2 variable selection methods = 8 computational experiments). All experimental models were run with 5-fold cross-validation.

Each Maxent model was formatted to run with logistic output to best conceptualize the output as estimates of the probability of suitability between values of 0 (unlikely to be present) and 1 (likely to be present). The number of background sample points varied for each population extent after buffering the species model training extent to have at least 10,000 background points (exact = 10,031): The southern, central, and northern models had 22, 30, and 48 occurrence points respectively and were modeled with 4,112, 4,487, and 6,143 background points, respectively–values that are proportional to the area of each population training extent (S1 Fig in S1 File). We set the maximum number of iterations to 500, though increased this number if all five cross validated replicates did not converge by 500. Finally, we applied a 10-percentile training presence threshold rule to obtain binary output to test our hypotheses. This threshold rule is conservative and finds the suitability value at which 10% of the training presence points are predicted absent (i.e., omission error) and uses it to reclassify pixels with suitability values below that value as unsuitable (absent) and above as suitable (present). It should be noted that we have different sample sizes for each population, which does introduce variability in sizes of training and testing subsets.

Additionally, because many niche studies rely solely on bioclimatic variables [41, 42], we include 8 additional computational experiments in the supplemental materials which repeat the methods described in our main manuscript, but built with only bioclimatic variables (i.e., excluding the four NHDPlus hydrological variables; S2 Table in S1 File). This is included to provide another dataset that makes comparisons between predictions based on solely bioclimatic variables vs. ecosystem species-relevant variables.

#### Ensemble modeling

We considered how the results of this single-algorithm approach, described above, compare to an ensemble (multiple-algorithm) approach. We built ensemble models from model agreement with five additional modeling algorithms. Each model was built using the training-testing occurrence data split from the respective Maxent “best” replicate run. The five additional algorithms used are appropriate for presence-background data: Bioclim [66], Domain [67], Mahalanobis Distance [68], Support Vector Machines [69–71] and Random Forest [72].

All models were run in RStudio with the packages “dismo” [73], “raster” [74], “randomForest” [75], and “kernlab” [76, 77]. As in Maxent, models were built with the same number of background points in the same geographic extents and were transformed into binary output using a 10% training sensitivity threshold. We maintained all models that performed with a test AUC greater than 0.7 for the final ensembles, which were calculated based on model agreement. In five of the sixteen cases, only one of these algorithms performed well-enough to keep. In three of the sixteen cases, three algorithms were kept, and in eight of the sixteen cases, four of the five algorithms were kept (details can be found in S4 Table in S2 File). We present results from Maxent models, but comparisons of ensemble model results to Maxent model results are presented for hypothesis 1 in S5 Table in S2 File and for hypothesis 2 in S6 Table in S2 File.

### Geographic extents

**Fig 1:**
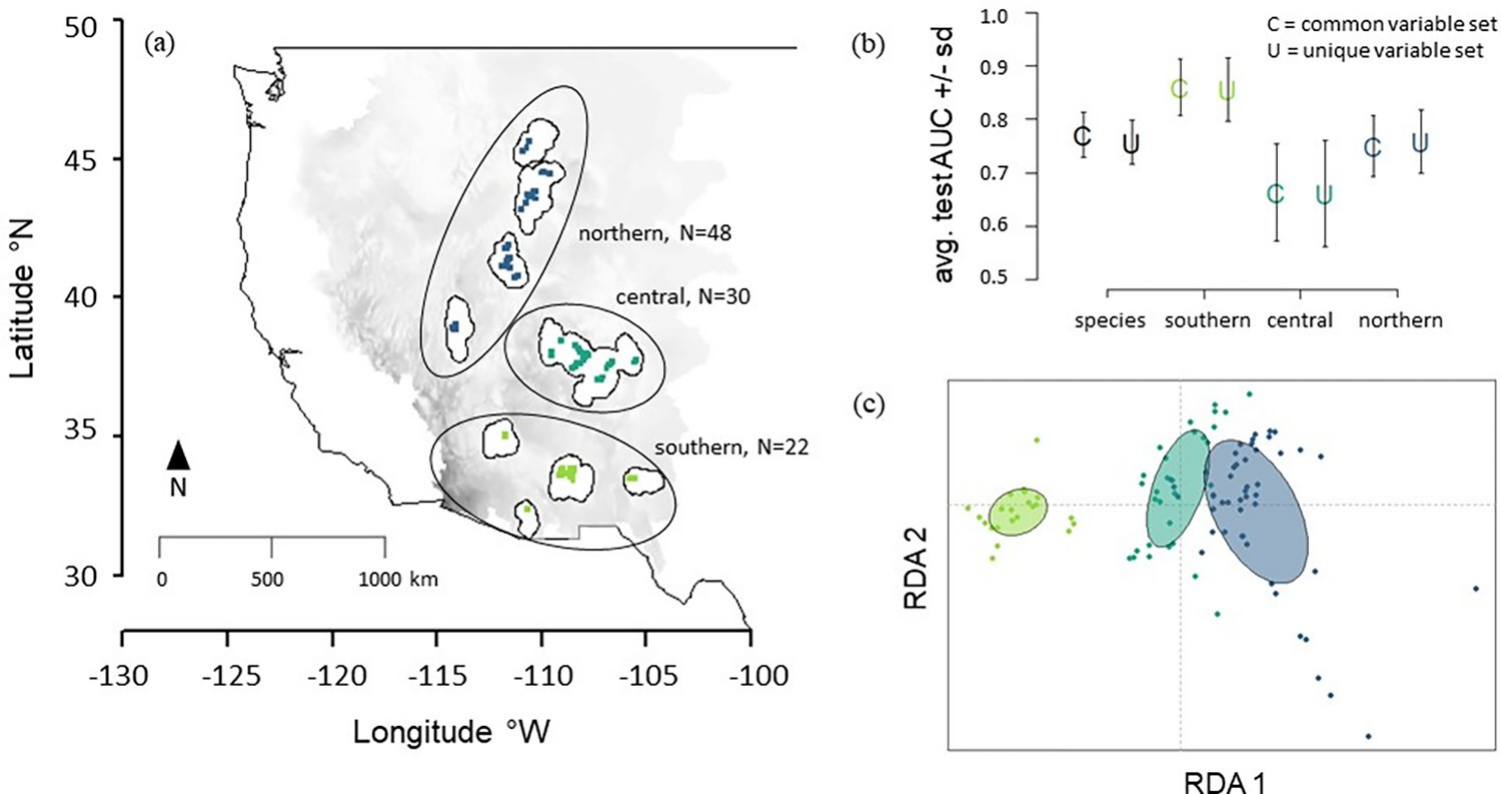
Panel (a) Geographic training extents and occurrence points. Panel (b) Test area under the curve of the receiver-operating characteristic values (AUC) +/- standard error for five cross-validated model replicates. Panel (c) RDA (Redundancy Analysis) plot of environmental variables included in final computational experiments. Geographic training extents are represented in white with corresponding occurrence records separated by color. The northern population is represented in dark blue (N = 48 occurrence records), the central population in teal (N = 30), and the southern population in green (N = 22). The background gradient represents the larger geographic space on which models are projected in the Western United States. In panel (b), color represents geographic training extent of model as in panel (a). Symbol (U or C) represents the variable selection method with “U” = unique predictor variable selection within respective geographic training extents and “C” = a common set of variables provided to all models. Details of “U” and “C” selection can be found in the methods. The background map of the United States represented in this figure was accessed through the R package “maptools” [82]. Maps are projected in WGS84 (World Geodetic System 1984) or EPSG 4326.

For training models, we created four occurrence datasets based on different geographic extents (regions): one was based on all occurrence points (“species”; N = 100) and three were based on these 100 occurrence points split into three genetic populations (southern, N = 20; central, N = 30; and northern, N = 48; Fig 1A, S1 Fig in S1 File). Based on assumptions about riparian dispersal for *P*. *angustifolia*–riparian network connectivity is related to genetic connectivity of *P*. *angustifolia* [55, 56]–we defined the exact extent of each training region by creating geographic bounds around occurrence points by mapping HUC (hydrologic unit code) level 8 from the USGS Watershed Boundary Dataset (https://water.usgs.gov/GIS/huc.html), which allowed us to include all relevant occurrence data points within the lowest number of water basins. The Watershed Boundary Dataset breaks hydrological regions into nested water basins of successively smaller hydrological units–the HUC code describes the “size” of the watershed designation with the largest level of classification having the United States divided into 21 major geographic regions. We accessed these data in RStudio [78] using the packages “nhdplusTools” [79] and “nhdR” [80; USGS] with the functions “download_wbd” and “get_huc8.” The combined HUC8 area of the species range was 94,543.22 square kilometers and includes land area in the states of New Mexico, Colorado, Utah, Nevada, Arizona, Wyoming, Idaho, and Montana. This total species’ range area split into 25,598.86 (southern), 32,087.01 (central), and 36,857.35 (northern) square kilometers areas for the genetic population ranges (Fig 1A, S1 Fig in S1 File). We added a geographic buffer to the HUC8 water basins to get final training extents. We did this in RStudio with the “buffer” function in the package “raster” [74] set to add a 25 km geographic buffer around the water basins, chosen so that no *entire* neighboring HUC8 regions would be included in the training region (this buffer is slightly smaller than recommended [81]; S1 Fig in S1 File).

### Environmental variable selection

We compare predictions built from models that were trained on “unique” sets of predictor variables to those built on a “common” set of predictor variables to understand the differences in niche overlap and geographic predictions that arise from different variable selection methods–both approaches allow population models to respond to environmental conditions differently and thus address the possibility of local adaptation. In one approach (“common”), variables are allowed to contribute differently to each model while in the other approach (“unique”) variables are chosen that may singularly important in the respective region.

“Unique” variable combinations were chosen by initial runs of 5-fold cross-validation Maxent models within each training extent. From the initial pool of variables included in the models, we selected those which cumulatively contributed between 90–95% to the gain in model fit of the five replicates for each group and removed any variables with high spatial correlation (0.70 used as the threshold) within the respective training extent. The “common” set of variables was chosen using the R package “embarcadero” (Version 1.2.0.1003) with the function “variable_step” [78, 83]. We specified the function to run 50 iterations of 10 trees (Bayesian Additive Regression Trees) within each of the four geographic training extents. The function eliminates variables with the lowest importance before recommending models with the lowest root mean square error (RMSE) [78, 83]. We combined the variables selected this way into a common pool used to build models for each training extent. After variable selection for all experimental models, each was run a final time again with 5-fold cross-validation.

### Model evaluation

We evaluated model performance using Area under the curve (AUC) of the receiver-operating characteristic, omission rate, and the Boyce Index. AUC is a commonly used threshold-independent metric of discriminatory ability—i.e., how accurately individual occurrences were predicted by the models. Higher values indicate lower type II error and values range from 0–1 with 0.5 representing random attribution of points [30]. To counter a known shortcoming that AUC is dependent on species prevalence [84], we also calculated omission error, or false-negative rates, that relies on user-specified thresholds which we specified as a 10% training omission error, described above in the modeling approaches section [85]. This threshold was used with training data to convert model suitability values to binary predictions. Finally, we calculated the Boyce Index to evaluate model performance. Like AUC, the Boyce Index is a threshold-independent metric. It is well suited for evaluating presence-only models that describes how much the model predictions differ from a random distribution of known presences across prediction gradients [86, 87]. High positive values of the Boyce Index indicate models with a stronger correlation between predicted:expected frequencies of test points with landscape suitability values [86]. These values were calculated in RStudio using the function “ecospat.boyce” from the R package “ecospat” (version 3.2) [78, 88].

Small sample sizes can affect the reliability of evaluation scores described above including the commonly used test AUC [89]. To test whether our models were better than random, we followed the approach of Bohl et al. 2019 [90]. This approach allows for comparison of the “real” niche models evaluation metrics to a distribution of evaluation metrics derived from null models. We compared each model’s test AUC to a null distribution of test AUC built from 1000 null niche models that were built from random subsamples of the background data used as calibration points. All null models were built with the same settings as the “real” Maxent models and were tested with the same evaluation occurrences used to test the “best-performing” Maxent replicate.

### Analyses

For all analyses and comparisons, we used the cross validation model replicate with the highest AUC value and lowest omission rate for each computational experiment.

#### Model comparisons in environmental niche space

To test if environmental variables used in models differed across the three genetically differentiated populations, we used redundancy analysis (RDA) with the R package “vegan” [78, 91] with population as the constrained axis. All variables were z-transformed prior to running models to reduce the effect of outliers and non-linear combinations. Results from the RDA explain how much of the total environmental variation can be attributed to population groupings–in other words, how different the background environmental variation is across populations.

We hypothesized that no single combination of environmental variables would be applied to genetically distinct populations of *P*. *angustifolia*. If the niche was conserved across populations, we would expect to select the same predictor variables for each population with the “unique” variable selection process, and/or that the “common” set of predictor variables would contribute similarly to each population model.

To compare the important variables for each genetic group we examined the percent contribution of environmental variables to Maxent experiments, and to compare niche overlap between models, we calculated Schoener’s D with ENMTools [33, 92, 93]. Schoener’s D provides a measure of niche overlap by comparing density distributions: The metric spans from 0–1 with values closer to zero indicating no overlap between models a value of 1 indicating full overlap of models [33, 92]. This metric provides a value between 0–1, where values closer to zero represent little overlap between modeled niches and a value of 1 representing full overlap of modeled niches.

#### Model comparisons in geographic space within population ranges and across the species’ range

To test our specific hypothesis that traditionally built species models would predict more suitable habitat within the ranges of genetic populations than individual models built from those groups, we cropped the species’ model predictions to each population’s training extent and compared the amount (%) of model agreement and disagreement in geographic space.

To test our hypotheses that genetic population models would predict more suitable habitat than species models across the Western United States, we projected all models across a larger region of the Western United States designated with water basins of the HUC4 level of the Watershed Boundary Dataset (USGS; described above). This region encompassed all training water basins (HUC8) across a continuous geographic region (region represented behind training regions as a background layer in Fig 1A; S1 Fig in S1 File). We merged all three population models into an “aggregated population model” and calculated the percentage of the landscape (the continuous HUC4-level geographic region) that was predicted suitable by any one, two, or three population models. This was compared to the species-level predictions to calculate the amount (%) of model agreement and disagreement in geographic space.

## Results

### Model evaluation

The test AUC of the best-performing cross validated replicates ranged from 0.66 (central common model) to 0.91 (southern unique model; Table 1, S5 Fig in S1 File). All models aside from the two central models had a test AUC > 0.8, indicating good discriminatory ability [94], and all models had average omission rates between 0.18 and 0.38 (Table 1, S5 Fig in S1 File). The Boyce Index for all models was positive, ranging from 0.688 to 0.919 (Table 1). Null distributions for all models can be found in S7-S10 Figs in S3 File.

**Table 1:** Description of cross-validated Maxent modeling experiments.

Description of Cross-Validated Maxent Modeling Experiments: Building, Evaluating, Performance							
	Training Extent	Avg. N train(test)	Avg. AUC_train_	Avg. AUC_test_	Max. AUC_test_	Avg. Omission	Boyce Index
				+/- Std. Dev.			
**unique (U)**	Species	80(20)	0.81	0.76 +/- 0.04	0.82	0.18	0.907
*predictor variables unique to each group*	Southern	17.6(4.4)	0.94	0.86 +/- 0.06	0.91	0.23	0.735
	Central	24(6)	0.78	0.66 +/- 0.10	0.67	0.20	0.871
	Northern	38.4(9.6)	0.88	0.76 +/- 0.06	0.86	0.29	0.869
**common (C)**	Species	80(20)	0.85	0.77 +/- 0.04	0.82	0.23	0.688
*predictor variables common across groups*							
	Southern	17.6(4.4)	0.95	0.86 +/- 0.05	0.89	0.36	0.919
	Central	24(6)	0.79	0.66 +/- 0.09	0.66	0.23	0.815
	Northern	38.4(9.6)	0.89	0.75 +/- 0.06	0.85	0.38	0.879

Description of Modeling Experiments includes sample size of training and testing data, average training AUC (Avg. AUC_train_), average test AUC +/- standard deviation (AUC_test_ +/- St. Dev.), test AUC of the highest performing model replicate (Max. AUC_test_), average omission rate of the five cross-validated replicates, and Boyce Index.

### Environmental niches of *P*. *angustifolia* vary across populations

We show that environmental variables vary on the landscape across the three genetically distinct populations of *P*. *angustifolia* with a redundancy analysis [78, 91]. This analysis revealed that 14% percent of the variance in all environmental response variables could be explained by the geographic extent of genetic population (p<0.001; Fig 1C). We ran two additional RDAs splitting the environmental data (all environmental variables included in models are in Table 2) into two predictor datasets: climate-only (ClimateNA variables) and hydrology-only (NHDPlusV2 variables). Genetic population extent explained 19.2% of the variance in the “climate-only” predictors (p<0.001) and only 2.3% of the variance in the “hydrology-only” predictor variables (p = 0.33).

**Table 2:** Percent (%) contribution of environmental variables to ENMs.

	unique: predictor variables unique to each group				common: predictor variables common across groups			
	Species	Southern	Central	Northern	Species	Southern	Central	Northern
**EREF:** Hargreave’s reference evaporation	**26.3**	-	**-**	**23.9**	**1.7**	**7.9**	**0.02**	**0.08**
**MAT:** mean annual temperature (°C)	**31.8**	-	**-**	**-**	**5.7**	**0**	**0**	**0.8**
**TD: **continentality (°C) MCMT -MWMT	**13.1**	**24.1**	**-**	**4.7**	**8.4**	**17.8**	**0.4**	**3.8**
**AHM: **annual heat moisture index (MAT+10)/(MAP/1000)	**-**	**30.9**	**-**	**-**	**13.6**	**9.2**	**6.0**	**1.2**
**MCMT:** mean temperature of the coldest month (°C)	**-**	**24.2**	**4.1**	**-**	**-**	**-**	**-**	**-**
**RH:** mean annual relative humidity (%)	**-**	**13.8**	**1.8**	**3.1**	**1.0**	**6.1**	**10.0**	**2.6**
**PAS:** precipitation as snow (mm)	**-**	**-**	**46.9**	**-**	**27.3**	**52.5**	**32.2**	**0.2**
**MSP:** mean summer (May to Sep) precipitation (mm)	**-**	**-**	**-**	**49.1**	**3.3**	**0.07**	**17.9**	**21.6**
**CMD:** Hargreave’s climatic moisture index	**-**	**-**	**-**	**-**	**21.6**	**0**	**0**	**42.5**
**NEAR:** distance to stream	**5.8**	**5.8**	**-**	**-**	**3.1**	**5.3**	**1.2**	**8.8**
**Q:** mean annual stream flow	**23.0**	**-**	**33.0**	**12.0**	**13.6**	**0.9**	**11.6**	**13.6**
**SO:** Stream Order	-	**1.1**	**14.2**	**7.2**	**0.9**	**0.1**	**20.6**	**4.7**

Blank cells indicate that the variable was not included in the model, while zeros indicate that the variable was included but contributed little to the final models. The top contributing variables for each model are bolded.

Significant environmental variation across the range of *P*. *angustifolia* (Fig 1C and S2 Fig in S1 File) provides an additional reason to examine environmental niches at the intraspecific level, and to carefully consider the types of environmental predictor variables included in models. We found no single combination of environmental variables that could be applied to genetically distinct populations of *P*. *angustifolia* (Table 2; i.e., through the “unique” selection process). Closer examination of important niche variables for the species and population models reveals how the environmental niches differ between groups. The result of the unique species model indicates that the niche of *P*. *angustifolia* is described largely by mean annual temperature (31.8%), Hargreave’s reference evaporation (26.3%) and mean annual stream flow (23%; Table 2). Mean annual stream flow is important to, or at least included in, all 7 models except the unique southern population model. The lowest contribution of mean annual stream flow was 0.9% in the common southern model (Table 2). Instead, for the southern-unique model, the hydrological variables combined contributed just about 7% (Table 2). The southern-unique niche was most well-described by annual heat moisture index (30.9%), mean temperature of the coldest month (24.2%), and continentality (the difference between mean temperature of the coldest month and the warmest month; 24.1%; Table 2). Precipitation as snow contributed largely to the unique central population model (46.9%). Mean summer precipitation (49.1%) and Hargreave’s reference evaporation (23.9%) contributed highly to the unique northern model (Table 2).

When a “common” set of variables was provided to models, the percent contribution of predictors varied greatly across models (Table 2). For instance, precipitation as snow (27.3%) contributed most to the species-common model with all other variables contributing between 0.9–21.6% (Table 2). Precipitation as snow also contributed highly 52.5% to the southern-common model, and Hargreave’s climatic moisture index contributed 42.5% to the northern-common model. Variable contributions were distributed from 0–32.2% to the central-common model (Table 2).

**Fig 2:**
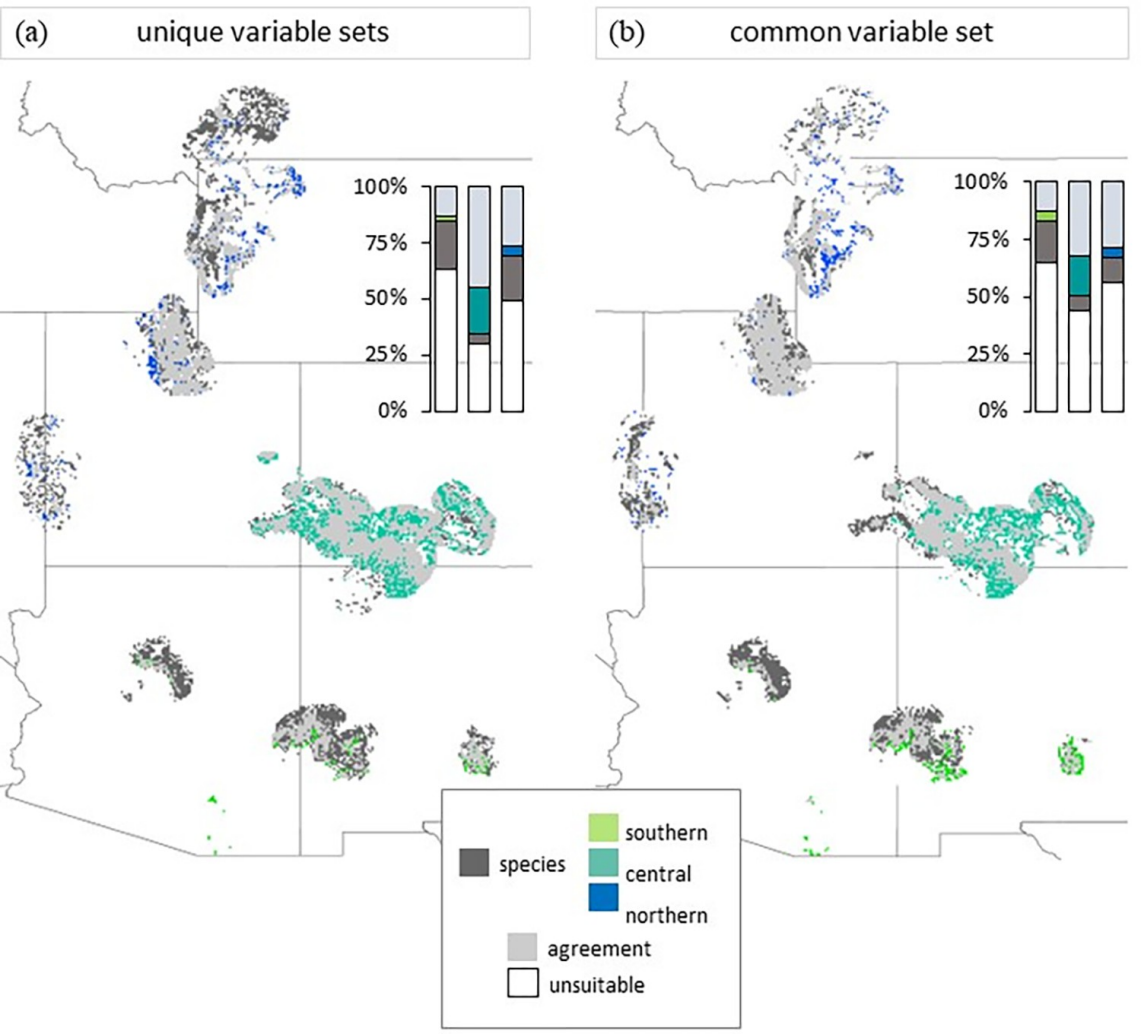
Geographic overlap of species predictions and population model predictions within the population geographic ranges. Panel (a) represents model comparisons built from “unique” variable sets and panel (b) represents model comparisons built from a “common” variable set. Inset stacked bar plots represent the percentage of the landscape within each population’s geographic range that was predicted as suitable habitat by the species model and each population model. Agreement between the model pairs on suitable habitat is represented in light grey on maps and bar plots. Though visualized together, calculations of overlap were calculated within each population extent: see S1 Fig in S1 File.

**Fig 3:**
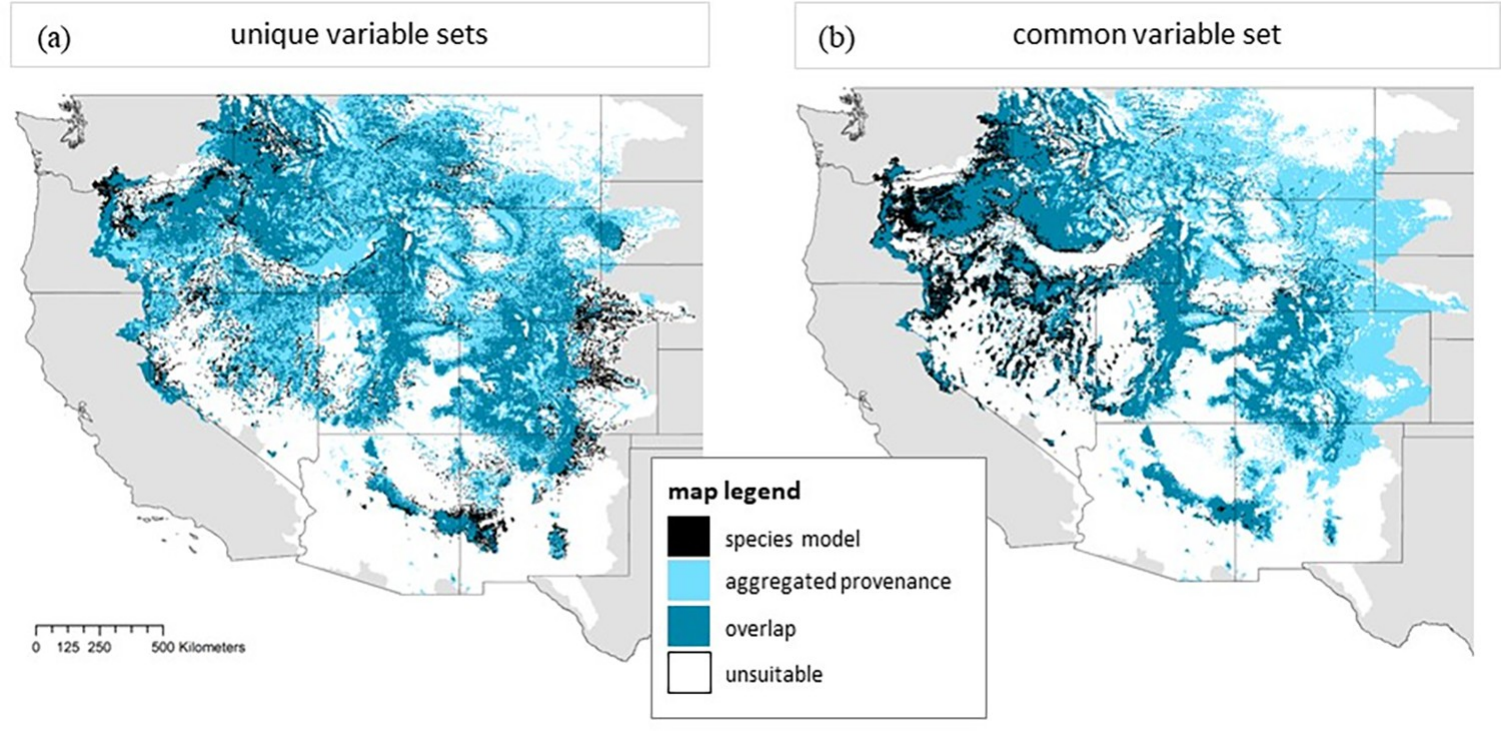
Maps representing the geographic overlap of aggregated population models with species models across the Western United States. Panel (A) uses models built with unique sets of predictor variables and panel (B) uses models built with a common set of predictor variables. Black regions represent suitable habitat predicted only by the species model, dark teal regions represent regions of model overlap or agreement on suitable habitat between the species model and the aggregated population models, and light blue represents regions predicted as suitable by the aggregated population model (at least 1 individual population model predicts suitable landscape in those locations). White regions are areas predicted unsuitable by all models. Maps are projected in WGS84 (World Geodetic System 1984) or EPSG 4326. The background map of the United States represented in this figure was downloaded from the U.S. Census (Cartographic Boundary Files (census.gov)).

The methodology of uniquely selecting environmental variables within populations compared to a common suite of selected variables did change predictions (Figs 2 and 3) and values of niche overlap (Schoener’s D; Table 3). The highest value of niche overlap was between the northern and species models and niche overlap was higher between these two models when built with common variables (0.910) than with unique variables (0.873; Table 3). Interestingly, the lowest value of niche overlap was between the southern and central populations for both variable selection types (Table 3).

**Table 3:** Niche overlap measured using Schoener’s D.

		Northern	Central	Southern
**unique (U) *predictor variables unique to each group***	**Species**	**0.873**	**0.865**	**0.764**
	**Northern**	**1**	**0.805**	**0.840**
	**Central**		**1**	**0.697**
	**Southern**			**1**
**common (C) *predictor variables common across groups***	**Species**	**0.910**	**0.864**	**0.793**
	**Northern**	**1**	**0.834**	**0.838**
	**Central**		**1**	**0.752**
	**Southern**			**1**

Values fall between 0 and 1, with values closer to zero representing no overlap between models while a value of 1 indicates full overlap of models.

Because many niche studies rely solely on bioclimatic variables [41, 42], we included 8 additional Maxent computational experiments in the supplemental materials built *only* with bioclimatic variables (i.e., excluding the four NHDPlus hydrological variables; S2 Table in S1 File) to compare to the model predictions from the eight models presented in the main manuscript. This additional comparison revealed that predictions can differ greatly (S6 Fig in S1 File) when these “species-specific” predictors are not included.

### Whether species-level model predicts more suitable habitat is region-specific

Within the geographic ranges of each genetically distinct population, species models predicted more suitable habitat than the respective population models in both the southern and northern extents, regardless of the variables used to build the models (common or unique; Fig 2). In the northern population extent, the two models agreed that 27% of the landscape was suitable and 49% was not when built with unique variables (Fig 2). Of the remaining percentage of the landscape, the species-model predicted 14.7% more suitable area (Fig 2). Though this overall pattern remained for the common variable comparison, the difference in predictions was just 7.4%. Overall, 6.6% less of the landscape was suitable to either model with shared “common” predictor variables. This geographic result also holds in environmental space where the niche overlap (Schoener’s D) is higher between the northern-species models when they are built with common variables than when they are not (Table 3).

The species model also predicted more suitable habitat within the southern population extent compared to the population model (Fig 2) and notably the smallest percentage of habitat was predicted to be suitable by either model in this region compared to others– 62.9% was unsuitable with the unique model comparison and 65.2% was unsuitable with the common model comparison (Fig 2).

In contrast to the southern and northern regions, within the central region, the population model predicted 15.5% more suitable habitat than the species model when both were built with unique sets of variables and 10.1% more suitable habitat when built with the set of common variables (Fig 2). Overall, this region had the least unsuitable habitat predicted (Fig 2).

### Geographic overlap across the species range

We found support for our hypothesis that the aggregated population models, regardless of predictor variable training sets, would predict more suitable habitat across the species’ range compared to the species-level model (Fig 3). With unique sets of predictor variables, the aggregated population model predicted 16.6% more of the landscape as suitable than the unique species-level model (Fig 3). The species model predicted suitable landscape on 32.9% of the landscape and the aggregated population model predicted suitable landscape on 49.5% of the landscape with predictions overlapping on 27.5% of the landscape. Models agreed that 45.2% of the landscape was unsuitable for *P*. *angustifolia*. This pattern of geographic overlap was similar with the common set of predictor variables, though overall 5.0% less habitat was predicted as suitable by either model type (species or aggregate population models; Fig 3) than with the unique models. The difference between model predictions of suitable habitat was 15.2% with the set of common predictor variables (Fig 3).

The aggregate unique population model predicted 6% more suitable habitat than the aggregate common population model, and the unique species model predicted 5.2% more suitable habitat than the common species model (Fig 3).

## Discussion

Significant environmental variation across species’ ranges begs a more deliberate identification of bioclimatic and ecological niches and a better understanding how, why, and when to include different types of variables in models. These results have broad implications for predicting population-level ecological and evolutionary responses to climate change. For example, the models presented in this study make different geographic predictions of suitable landscape when built with unique sets of variables selected at a population-level compared to when built with common sets of variables selected at the species-level. Importantly, this emphasizes the need to continue challenging the assumption of distribution modeling that a species is a uniform unit across its’ geographic range–in other words, ignoring the fact that populations are likely locally adapted to unique environmental conditions. Additionally, we show the importance of including species-specific predictor variables in addition to bioclimatic predictors–in the case of our riparian study species, predictions of suitable habitat should be confined to riparian zones.

Overall, our study adds to accumulating evidence [10, 12, 13, 19] that building ENMs at the species-level can lead to misleading predictions for certain populations and genetic populations in the face of climate change. The lack of a “single niche” for the species emphasizes the large extent to which improvements depend not only on the inclusion of genetic information but also on the type and selection of predictor variables that interact with that genetic information. Many recent models attempt to assess whether including genetic information into distribution or niche models increases model accuracy [52], but this is a difficult methodological question to assess when models are built in different geographic areas, with different numbers of occurrences, and with different numbers of predictor variables–for example, range size, sample size, and the number of predictor variables can affect model accuracy, namely AUC values [95, 96].

### There is no single species-level niche that can be applied to populations

As expected, the southern population showed the lowest environmental niche overlap with the species-wide model (Table 3). The species-level models predicted more suitable habitat within population ranges for two of the three genetic groups regardless of variable selection method, but it was within the “central” population extent where this was not the case. This could be because this region captures either a larger range of environmental conditions in the core of the species’ range and/or less extreme environmental conditions than the other two regions (S2 Fig in S1 File).

We cannot conclude that genetic population ENMs trained on a unique set of environmental variables (selected within each population’s geographic bounds) performed better than ENMs were provided with the common set of predictor variables because the “common” models included a higher number of predictor variables which inherently can increase model performance metrics (Table 1). However, model performance was good overall across models (Table 1, S7-S10 Figs in S3 File), suggesting that it may instead be important to consider in which scenarios different variable selection methods may be more useful. For example, it may be more useful to select unique variable sets at the genetic population level when projecting models across space or time, given that the identity and contributions of variables differed considerably from the common selection and across genetic groups (Table 2) as these differences may better reflect variation in population tolerances to environmental conditions. This idea is also reflected in the result that aggregate unique population model predicted more suitable habitat than the aggregate common population model, and the unique species model predicted more suitable habitat than the common species model (Fig 3; S4 Fig in S1 File).

Our study also stresses the importance of considering “species-relevant” predictor variables [36]. While again we hesitate to make conclusions about these variables improving model performance (e.g., conclusions that are often inaccurately draw from figures like S5 Fig in S1 File), we do stress that including these variables make very different predictions of suitable habitat on the landscape (S6 Fig in S1 File). This is a critical as most models assume that climate is the main driver of species distributions at a large scale [41, 42]. Though climate variables can typically describe a species’ current range [43], they may limit the accuracy of predictions across space or time, where species-specific predictor variables may improve these predictions [34, 40, 42, 43]. Though the redundancy analysis split by variable type showed that hydrological “species-specific” predictors explain less variation than climate predictors across the genetic population ranges, the difference in predictions (S6 Fig in S1 File) seem particularly important for a riparian species where proximity to stream water may offset some climate stress associated with heat or precipitation.

### Species-level models do not always predict broader suitable habitat within population regions

Overall, species-range ENMs did not always predict more suitable habitat within the geographic ranges of genetic populations (Fig 2). We hypothesized this because the species models would be trained on a larger range of environmental conditions than the population models. More broad predictions were expected from range-wide models as a species’ range spans broader environmental gradients and larger areas than intraspecific delineations [2, 27]–especially when the common suite of environmental predictor variables were used to build models. However, it was only in the “central” population range where the species model did not predict more suitable habitat. The species model did predict more suitable habitat in the southern and northern population ranges and the exact amount increased when the models were built with a common set of variables. This suggests that the more “extreme” ends of the range are influencing species’ model predictions at the opposite “extreme” or “edges” of the range. We predicted this would be the case because a species-model captures a larger range of environmental conditions that are tolerated by all populations. These results indicate that niches defined at a population-level may indeed better capture local adaptation to environmental conditions. If species models regularly over-predict suitable geographic distributions, then ENMs may be less likely to predict risk or response of populations to global change factors.

### Aggregated population models predict more suitable habitat across the species’ range

When combined into a single output, the aggregated genetic population models predicted broader suitable distribution over the entire species’ range than the species’ models regardless of variable selection method (Fig 3). This suggests that niche variables defined at the population-level may capture local adaptation to environmental conditions and allow for more refined predictions of population responses to environmental change. This supports recent findings that lineage-level predictions predict broader suitable habitat than species-level predictions [13].

## Conclusions

Incorporating intraspecific variation into ecological niche models (ENMs) has been hypothesized to increase model accuracy, change estimates of risk of species-level declines, and reveal differential responses of intraspecific groups to climate change depending on range-position (e.g., edge vs. central lineages) and/or performance-climate relationships (e.g., warm-adapted lineages). Overall, this study emphasizes the need to consider how and why environmental variables are selected for ENMs, *especially* when including genetic substructure within a species. More nuanced ENMs should allow for a more refined understanding of species and population-level risks in the face of climate change. Our findings also advance current understanding of how distribution models should be interpreted and used. For instance, these results add to accumulating evidence that a major assumption made by traditional species distribution (or ecological niche) models that populations will all respond similarly to climate change is often violated. This advance in our understanding of these models should be useful to conservation managers who need accurate predictions to better identify where, and which, populations, communities and ecosystems are most at risk due to climate change. Decisions should not depend on models built solely from species-wide ranges.

## Supporting information

S1 File Includes S1-S3 Tables and S1-S6 Figs. (PDF)

S2 File Includes S4-S6 Tables related to ensemble model–Maxent model comparisons. (PDF)

S3 File Includes S7-S10 Figs showing null Maxent model distributions. (PDF)
