# Supplementary material for: Aggregate population-level models informed by genetics predict more suitable habitat than traditional species-level model across the range of a widespread riparian tree
Source: PLoS One. 2022 Sep 19;17(9):e0274892. doi: 10.1371/journal.pone.0274892 (PMC9484645; doi:10.1371/journal.pone.0274892)

Supporting Information File 1

S1_File

Includes: S1-S3 Tables and S1- S6 Figs

S1 Table: Occurrence records. Group = genetic population (southern, central, northern); X= longitude; Y= latitude.

group	X	Y
southern	-111.743	34.99061
southern	-111.737	35.02451
southern	-111.72	35.06177
southern	-110.695	32.37244
southern	-109.141	33.59392
southern	-109.072	33.69767
southern	-109.071	33.75107
southern	-109.055	33.59392
southern	-109.045	33.74882
southern	-109.025	33.66733
southern	-109.011	33.82554
southern	-108.769	33.67565
southern	-108.593	33.5299
southern	-108.542	33.53751
southern	-108.487	33.40886
southern	-108.462	33.7772
southern	-105.78	33.46554
southern	-109.098	33.62738
southern	-109.088	33.66716
southern	-108.593	33.81724
southern	-108.462	33.81676
southern	-105.584	33.46405
central	-109.542	38.02569
central	-109.534	37.90141
central	-109.517	37.98118
central	-109.042	38.44421
central	-109.04	38.41409
central	-108.508	37.47039
central	-108.401	38.26471
central	-108.336	37.6327
central	-108.223	38.13169
central	-108.15	38.05665
central	-108.135	37.59306
central	-108.113	37.60045
central	-108.021	37.74096
central	-107.988	37.76814
central	-107.975	37.96899
central	-107.883	37.84852
central	-107.797	37.93516

central	-107.318	37.0166
central	-107.139	37.04876
central	-107.052	37.12634
central	-106.766	37.56558
central	-106.729	37.59261
central	-106.605	37.67793
central	-105.587	37.65343
central	-108.401	38.26643
central	-108.39	37.50961
central	-107.838	37.94892
central	-106.89	37.44247
central	-106.604	37.67886
central	-105.518	37.73735
northern	-114.23	38.92245
northern	-114.208	38.85969
northern	-114.163	38.91166
northern	-114.123	39.01197
northern	-114.103	38.85393
northern	-114.084	38.92325
northern	-111.906	41.13595
northern	-111.775	41.74183
northern	-111.731	41.74857
northern	-111.697	41.76302
northern	-111.666	41.27059
northern	-111.664	41.76827
northern	-111.62	41.81408
northern	-111.599	41.40054
northern	-111.529	41.41323
northern	-111.522	41.04441
northern	-111.52	41.04296
northern	-111.243	40.73841
northern	-111.113	40.78587
northern	-111.108	40.76122
northern	-111.103	40.74995
northern	-110.989	43.17745
northern	-110.754	43.38893
northern	-110.705	45.39885
northern	-110.676	43.70647
northern	-110.584	43.64041
northern	-110.583	43.64027
northern	-110.571	45.63492
northern	-110.566	45.59686
northern	-110.428	43.61089

northern	-110.308	43.82412
northern	-110.282	43.55255
northern	-110.271	43.81505
northern	-110.002	44.48992
northern	-109.933	44.49226
northern	-109.629	44.47101
northern	-109.568	44.46022
northern	-109.556	44.46021
northern	-114.229	38.92175
northern	-114.18	38.86516
northern	-114.133	38.91882
northern	-114.128	38.85812
northern	-111.756	41.10803
northern	-111.643	41.7809
northern	-111.59	41.3761
northern	-111.572	41.86426
northern	-110.869	45.25479
northern	-110.39	43.8343

S2 Table. Description of Modeling Experiments including additional eight computational experiments run with only bioclimatic data from ClimateNA (grey rows). Includes sample size of training and testing data, average training AUC, average test AUC +/- standard deviation, average omission rate, best replicate of the five cross-validated replicates, and AUC test of the highest-performing replicate.

**Variable Selection Approach**	**Predictor Variables Included**	**Population/ Geographic Extent**	**Average AUC_{train}**	**Average AUC_{test}**	**+/- sd**	**Average Omission Rate**	**Best Replicate**	**Best Replicate AUC_{test}**
**unique** (U) *predictor variables unique to each group*	Both Variable Types	Species	0.81	0.76	0.04	0.18	_3	0.82
		Southern	0.94	0.86	0.06	0.23	_3	0.91
		Central	0.78	0.66	0.10	0.20	_0	0.67
		Northern	0.88	0.76	0.06	0.29	_1	0.86
	Climate-Only Variables	Species	0.75	0.71	0.05	0.13	_1	0.74
		Southern	0.93	0.867	0.06	0.28	_3	0.93
		Central	0.77	0.67	0.09	0.17	_0	0.77
		Northern	0.85	0.78	0.06	0.34	_0	0.82
**common** (C) *predictor variables common across groups*	Both Variable Types	Species	0.85	0.77	0.04	0.23	_2	0.82
		Southern	0.95	0.86	0.05	0.36	_3	0.89
		Central	0.79	0.66	0.09	0.23	_1	0.66
		Northern	0.89	0.75	0.06	0.38	_1	0.85
	Climate-Only Variables	Species	0.82	0.77	0.04	0.18	_2	0.81
		Southern	0.94	0.87	0.05	0.32	_3	0.95
		Central	0.77	0.68	0.09	0.2	_0	0.74
		Northern	0.86	0.76	0.06	0.31	_1	0.83

S3 Table. Niche overlap measured with Schoener's D. Rows in white are the same models presented in the main manuscript Table 3, presented here for ease of comparison to the climate-only models (grey rows). Values fall between 0 and 1, with values closer to zero representing no overlap between models while a value of 1 indicates full overlap of models.

Variable Selection Approach	Geographic Extent/ Population	Both Variable Types			Climate-Only Variables		
		Northern	Central	Southern	Northern	Central	Southern
**unique (U)** *predictor variables unique to each group*	Species	0.873	0.865	0.764	0.766	0.846	0.678
	Northern	1	0.805	0.840	1	0.829	0.836
	Central		1	0.697		1	0.739
	Southern			1			1
**common (C)** *predictor variables common across groups*	Species	0.910	0.864	0.793	0.881	0.854	0.75
	Northern	1	0.834	0.838	1	0.799	0.839
	Central		1	0.752		1	0.690
	Southern			1			1

S1 Fig. Geographic training extents. Four training extents built from the Watershed Boundary Dataset HUC8 basins + 25km buffer for panel (a) species, panel (b) southern, panel (c) central, and panel (d) northern regions. The geographic extent used for projecting models (HUC4 basins) is represented by a background layer on the map in panel (a).

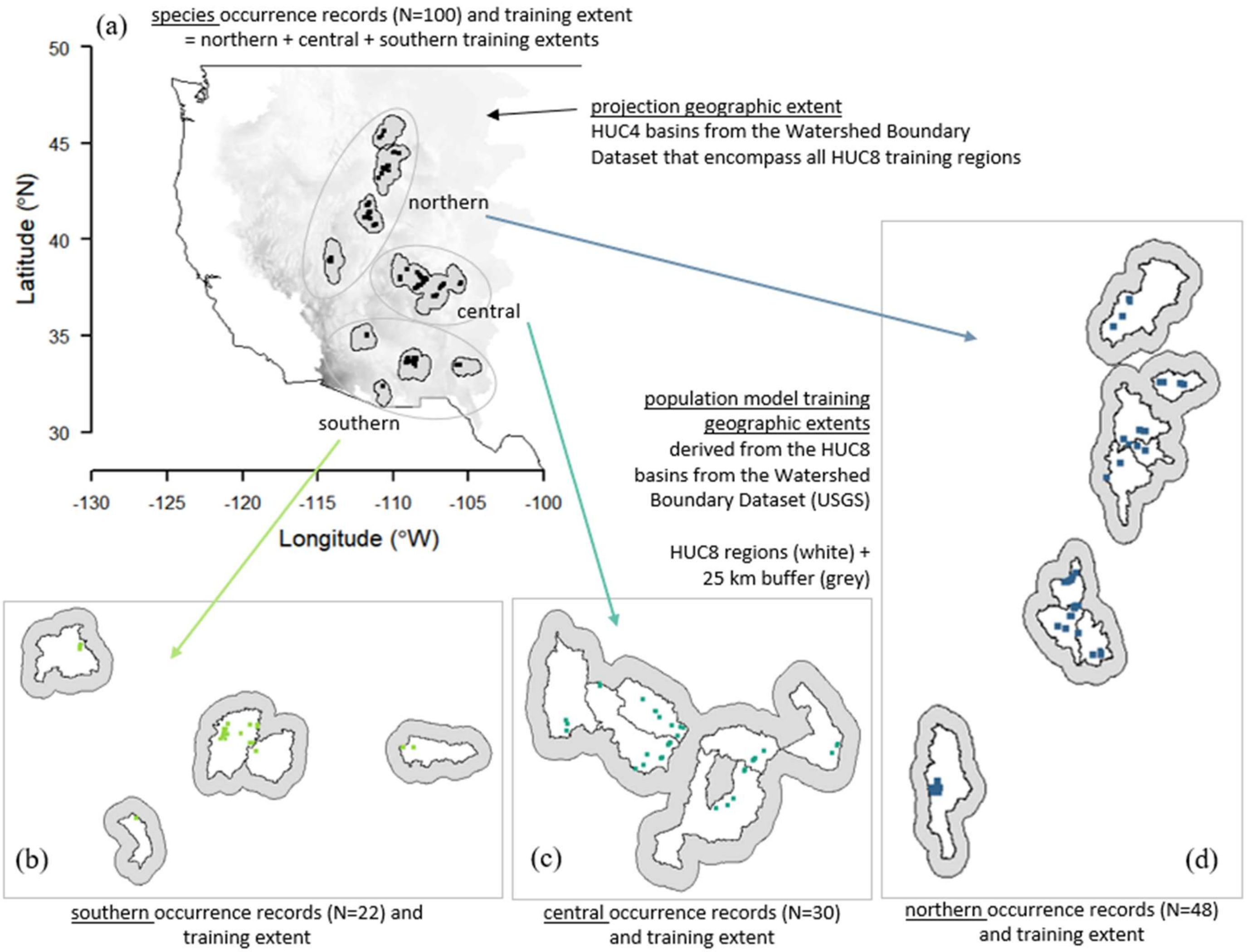

S2 Fig. Ecological niche model predictor variables across genetic groups. The x-axis labels p1, p2, and p3 refer to the southern, central, and northern populations, respectively. Variables are the same as those represented in Table 2 in the main manuscript, and include (top left to bottom right): Annual heat moisture index calculated as (mean annual temperature + 10) / (mean annual precipitation/1000), Hargreave's reference evaporation, mean annual precipitation (mm), mean annual temperature (C), mean temperature of coldest month (C), mean summer (May-September) precipitation (mm), precipitation as snow, continentality calculated as mean temperature of the coldest month - mean temperature of the warmest month, distance to nearest stream, mean annual stream flow, and mean stream order.

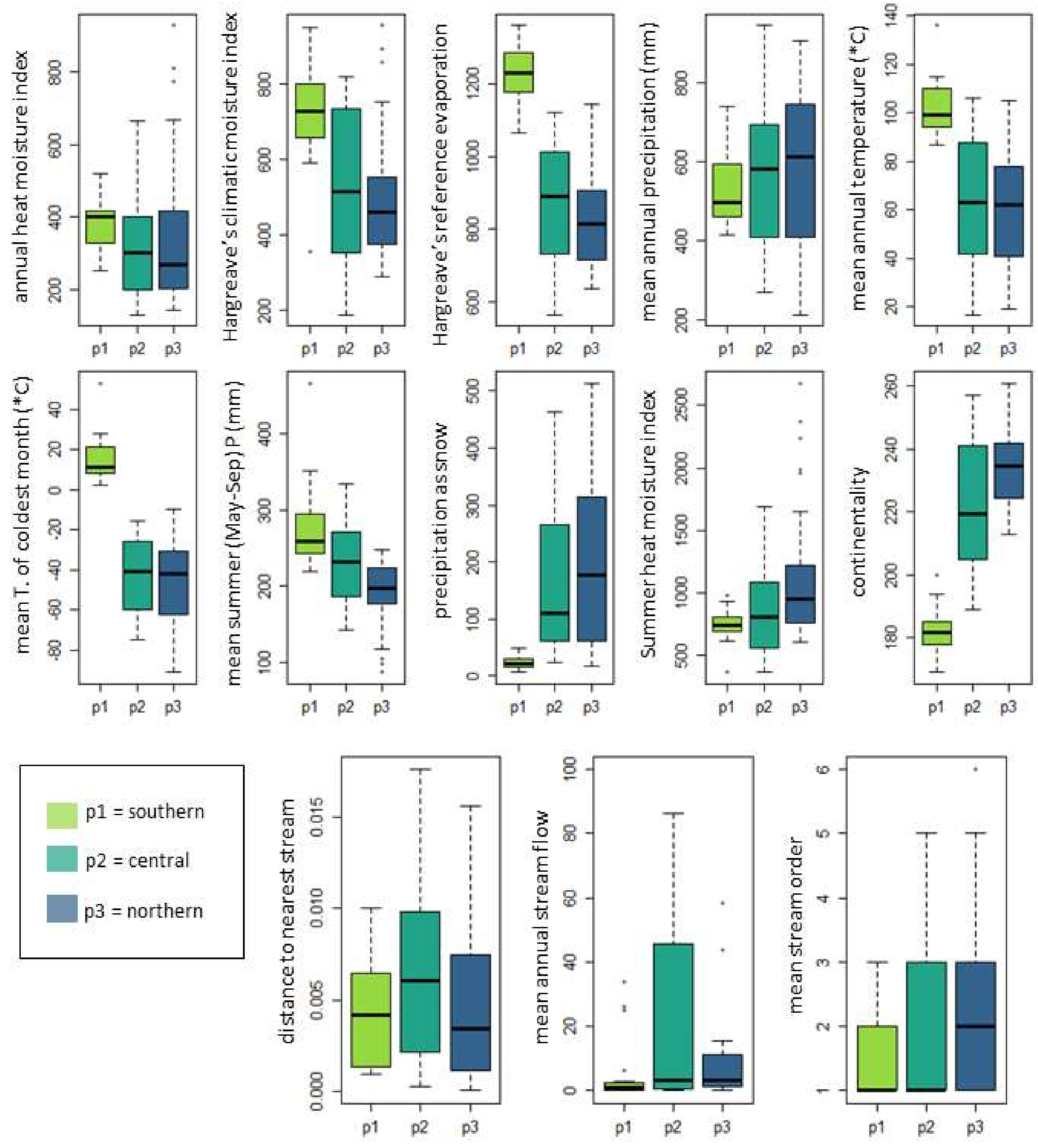

S3 Fig. Maps of species-level and individual population-level predicted overlap of suitable landscape within population range extents. The top row shows the same maps and barplots presented in Fig. 2. The bottom row shows the corresponding map overlap and barplots for models built with only climate predictors. Inset stacked barplots represent the percentage of the landscape within each population's geographic range that was predicted as suitable habitat by the species model and each population model. Agreement between the model pairs on suitable habitat is represented in light grey on the maps and in the barplots.

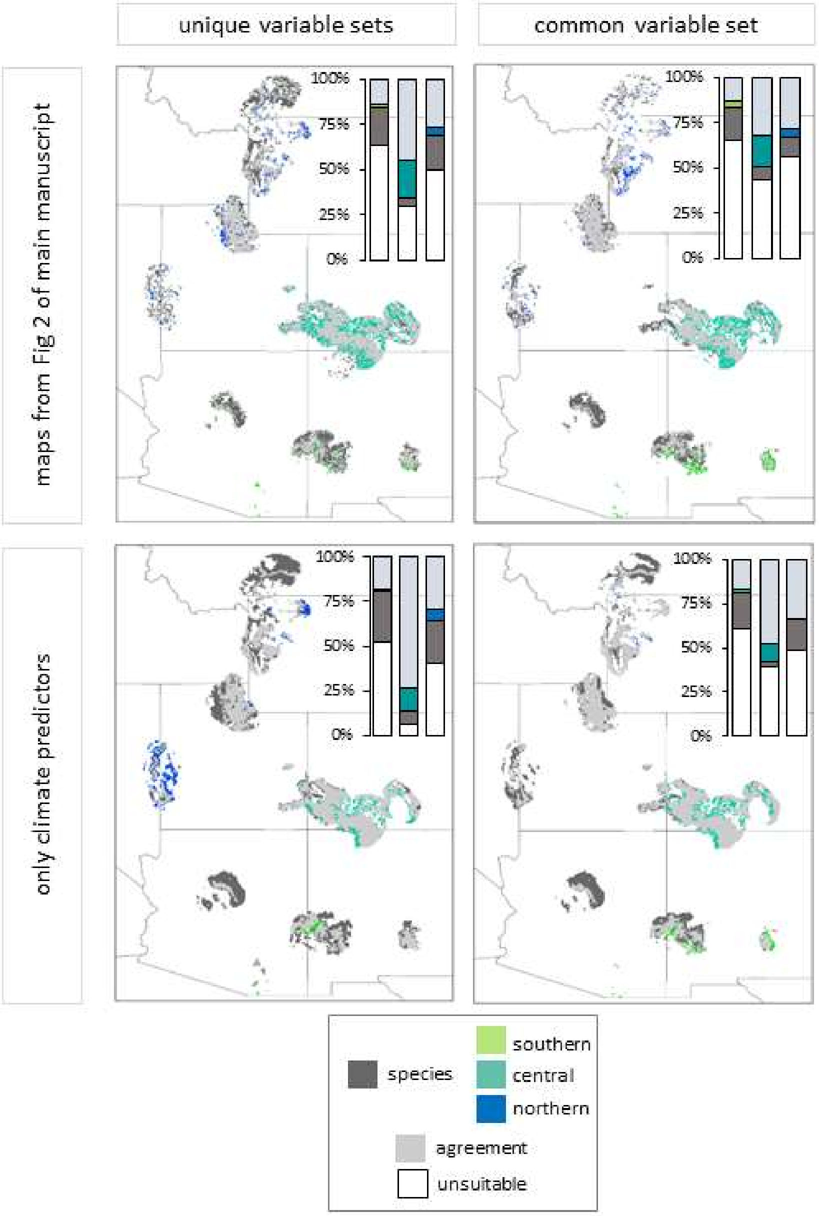

S4 Fig. Maps representing the geographic overlap of aggregated population models with species models across the Western United States. Black regions on the map represent suitable habitat predicted only by the species model, light blue represents regions predicted as suitable by the aggregated population model (at least 1 individual population model predicts suitable landscape in those locations), and dark blue regions represent regions of model overlap or agreement. White regions are areas predicted unsuitable by all models.

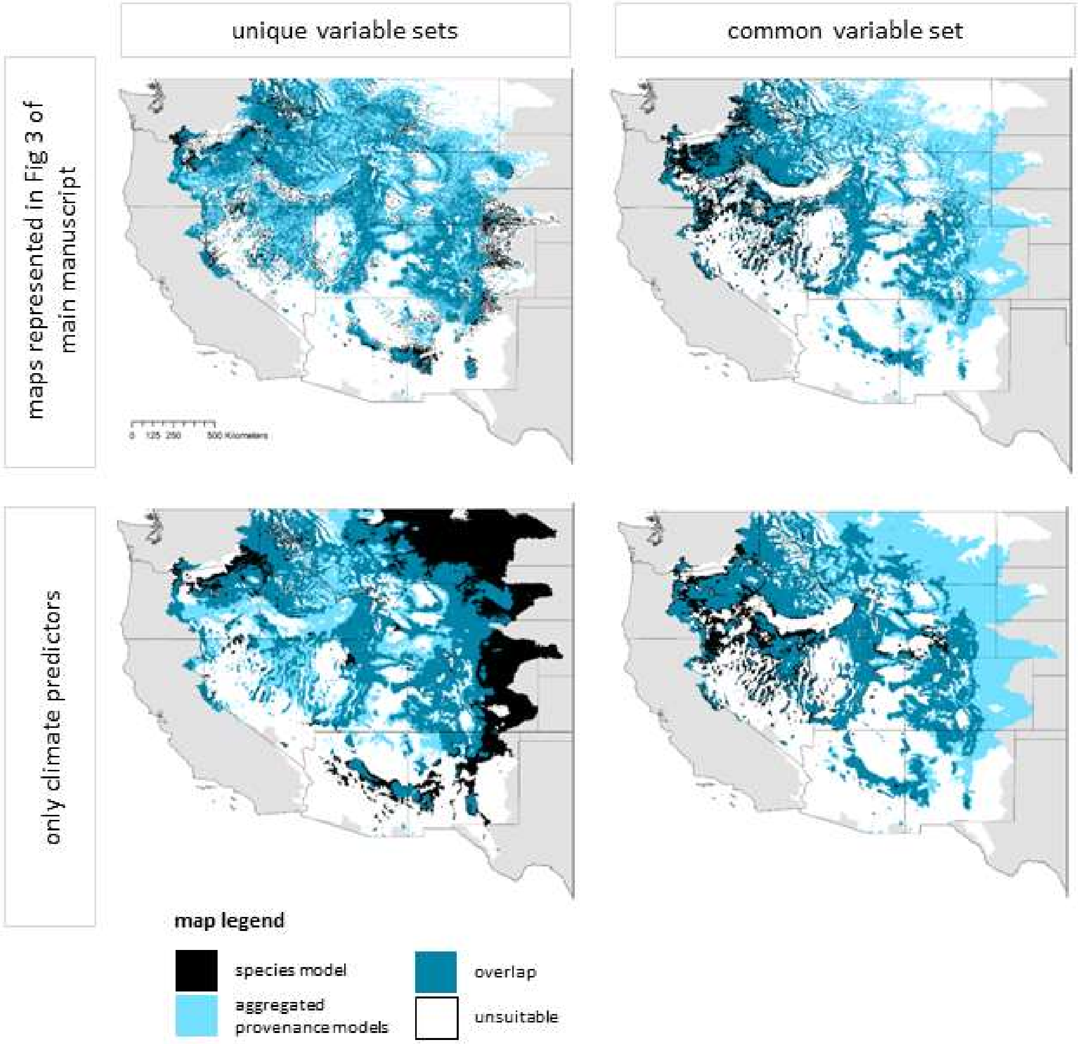

S5 Fig. Test area under the curve of the receiver-operating characteristic values (AUC) +/- standard error for five cross-validated model replicates, and average omission rate. Data are presented with additional climate-only computational experiments. The x-axis represents the model's geographic training extent (as in Fig 1 and S1 Fig: species, southern, central, or northern) and the environmental predictor variable set ("U" for unique and "C" for common). Additional climate-only models are represented by square symbols while models that were presented in the main manuscript are represented by circular symbols. Open symbols in the background represent the "best" performing model.

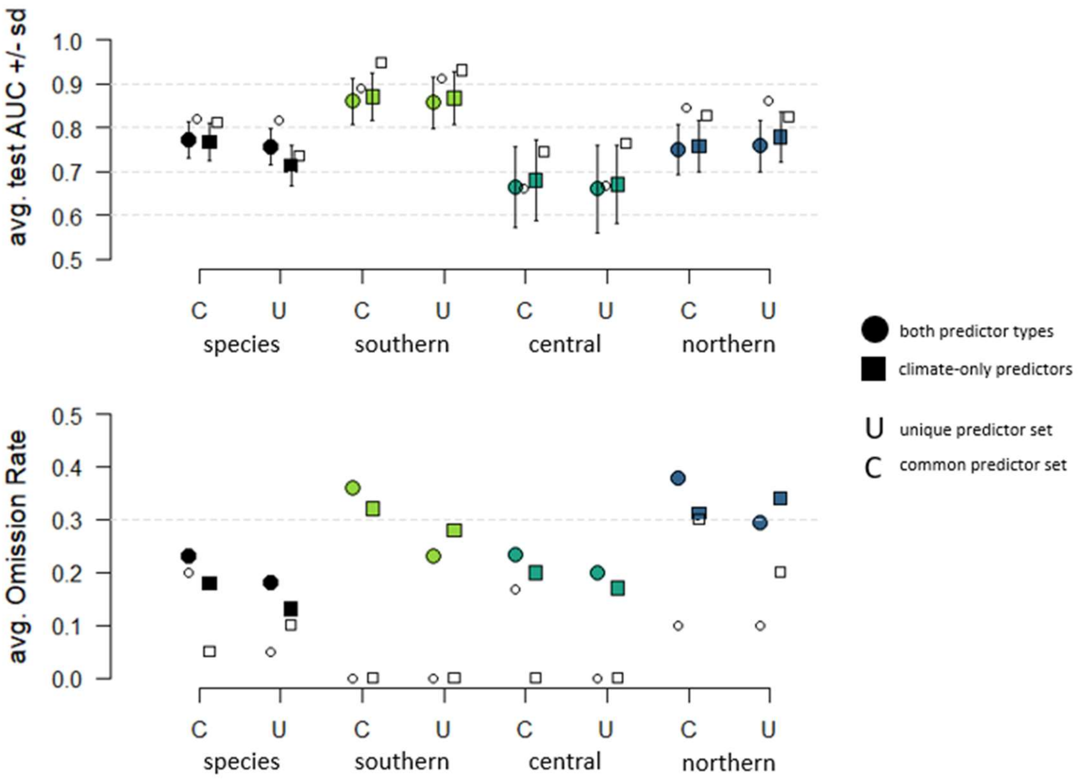

S6 Fig. Geographic overlap of models built with and without hydrological variables that are relevant to the environmental niche of *P. angustifolia,* a riparian species. Climate-only model predictions are represented in red and models with climate and hydrological variables (as included in the main manuscript) are represented in blue. Overlap is purple.

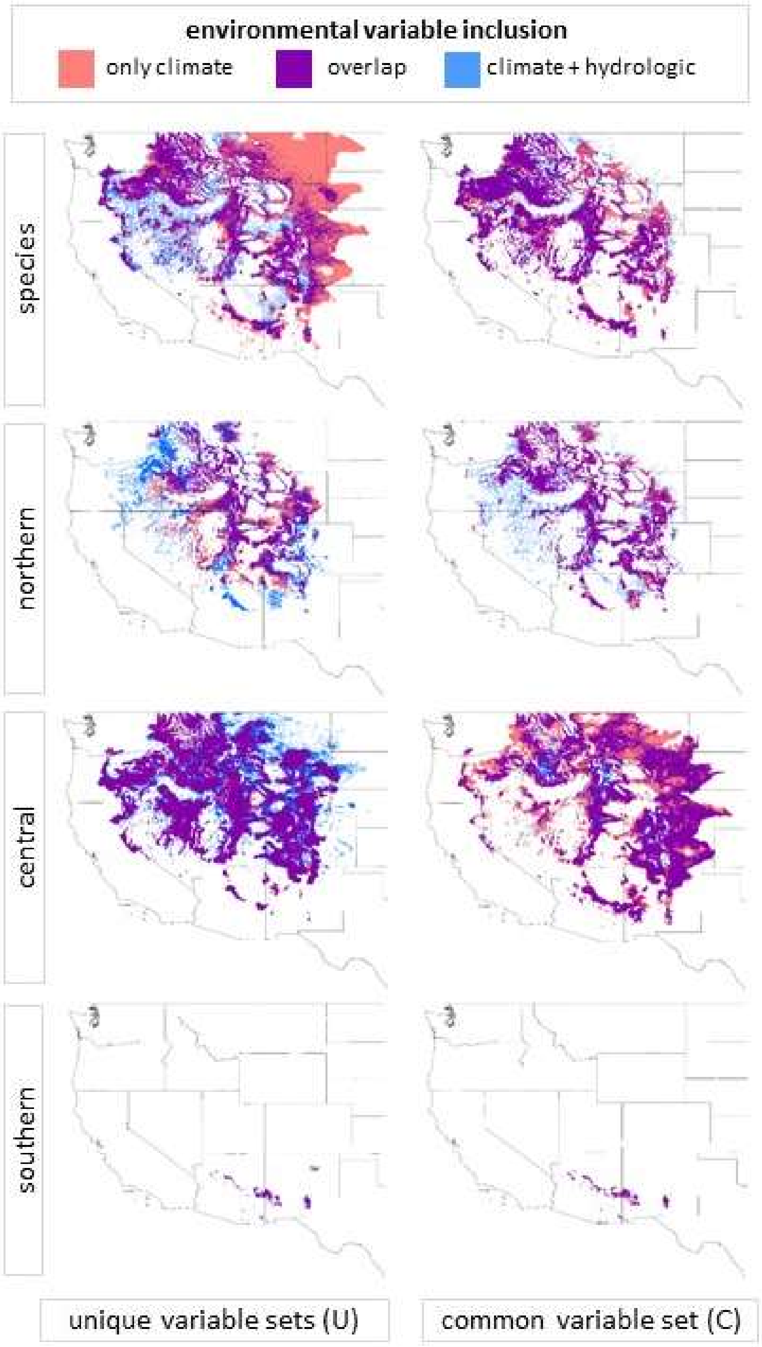

Supplement: S1 File — (PDF) [file pone.0274892.s001.pdf]
