# Supplementary material for: Aggregate population-level models informed by genetics predict more suitable habitat than traditional species-level model across the range of a widespread riparian tree
Source: PLoS One. 2022 Sep 19;17(9):e0274892. doi: 10.1371/journal.pone.0274892 (PMC9484645; doi:10.1371/journal.pone.0274892)
Supplement: S2 File — (PDF) [file pone.0274892.s002.pdf]

Supporting Information File 2

S2_File

Includes S4-S6 Tables

S4 Table. Summary of Ensemble Models. Table shows the Average test AUC for all five algorithms built for ensemble models, the number of modeling algorithms kept of the five, which of the modeling algorithms were kept for the final model, and the average test AUC of those that were kept for final ensemble model predictions. Modeling algorithms are abbreviated in the table as follows: B= Bioclim, D=Domain, M= Mahalanobis Distance, S= Support Machine Vectors, and R = Random Forest.

Variable Selection Method & Variable Type	Geographic Extent	Average test AUC (N= 5)	N algorithms kept	Which algorithms kept (> 0.7 test AUC) Abbreviations in caption.	Average test AUC (algorithms kept)
Common Variable Set & Both Variable Types	Species	0.73	4	B, M, S, R	0.76
	Southern	0.54	**1**	**Random Forest**	0.9
	Central	0.64	3	B, D, M	0.74
	Northern	0.77	4	B, D, M, R	0.83
Common Variable Set & Climate Only Variables	Species	0.75	3	M, S, R	0.82
	Southern	0.78	4	B, D, M, R	0.92
	Central	0.64	**1**	**Domain**	0.74
	Northern	0.76	4	B, D, M, R	0.83
Unique Variable Sets & Both Variable Types	Species	0.71	**1**	**Random Forest**	0.8
	Southern	0.67	3	B, M, R	0.81
	Central	0.66	**1**	**Bioclim**	0.78
	Northern	0.74	4	B, D, M, R	0.81
Unique Variable Sets & Climate Only Variables	Species	0.71	4	B, D, M, R	0.73
	Southern	0.87	4	B, D, M, R	0.92
	Central	0.63	**1**	**Bioclim**	0.77
	Northern	0.77	4	B, D, M, R	0.81

S5 Table. Ensemble-Maxent Comparison for Hypothesis 1. *Hypothesis: species-level models predict more suitable habitat within the ranges of genetic populations than individual models built from those groups.* Table shows the agreement between population models and species model predictions within the geographic training extents of each population. "Species > Population" means that the species model predicts more suitable habitat, and accordingly, "Population > Species" means that the population model predicted more suitable habitat than the species model.

Variable Selection Method	Variable Type	South - Species		Central - Species		Northern - Species	
		Maxent	Ensemble*	Maxent	Ensemble*	Maxent	Ensemble*
Unique	Both	**Species > Population**		**Population > Species**	**Species > Population**	**Species > Population**	
	Climate						
Common	Both						
	Climate			**Population > Species**			

*Please note that in five cases, the "ensemble" column heading is misleading. In these 5 cases, the "final ensemble" model is the output from a single modeling algorithm. These cases are (1) Southern Common-Both, (2) Central Common-Climate, (3) Species Unique-Both, (4) Central Unique-Both, and (5) Central Unique-Climate. Details can be found in Table on the previous page.

S6 Table. Ensemble-Maxent Comparison for Hypothesis 2. *Hypothesis: aggregated genetic population models predict more suitable habitat across the species' range than the species-level model.* Table shows the agreement between aggregate population models (suitable habitat predicted by any one population model) and species model predictions across the geographic range. "Aggregate Population > Species" means that the aggregated population model predicted more suitable habitat than the species model.

Variable Selection Method	Variable Type	Maxent	Ensemble
Unique	Both	**Aggregate Population > Species**	**Species > Aggregate Population**
	Climate	**Species > Aggregate Population**	
Common	Both	**Aggregate Population > Species**	
	Climate	**Aggregate Population > Species**	
