# Supplementary material for: Aggregate population-level models informed by genetics predict more suitable habitat than traditional species-level model across the range of a widespread riparian tree
Source: PLoS One. 2022 Sep 19;17(9):e0274892. doi: 10.1371/journal.pone.0274892 (PMC9484645; doi:10.1371/journal.pone.0274892)

Supporting Information File 3

S3_File

Includes S7-S10 Figs

S7 Fig: Null Distribution of Test AUC values for species-wide models.

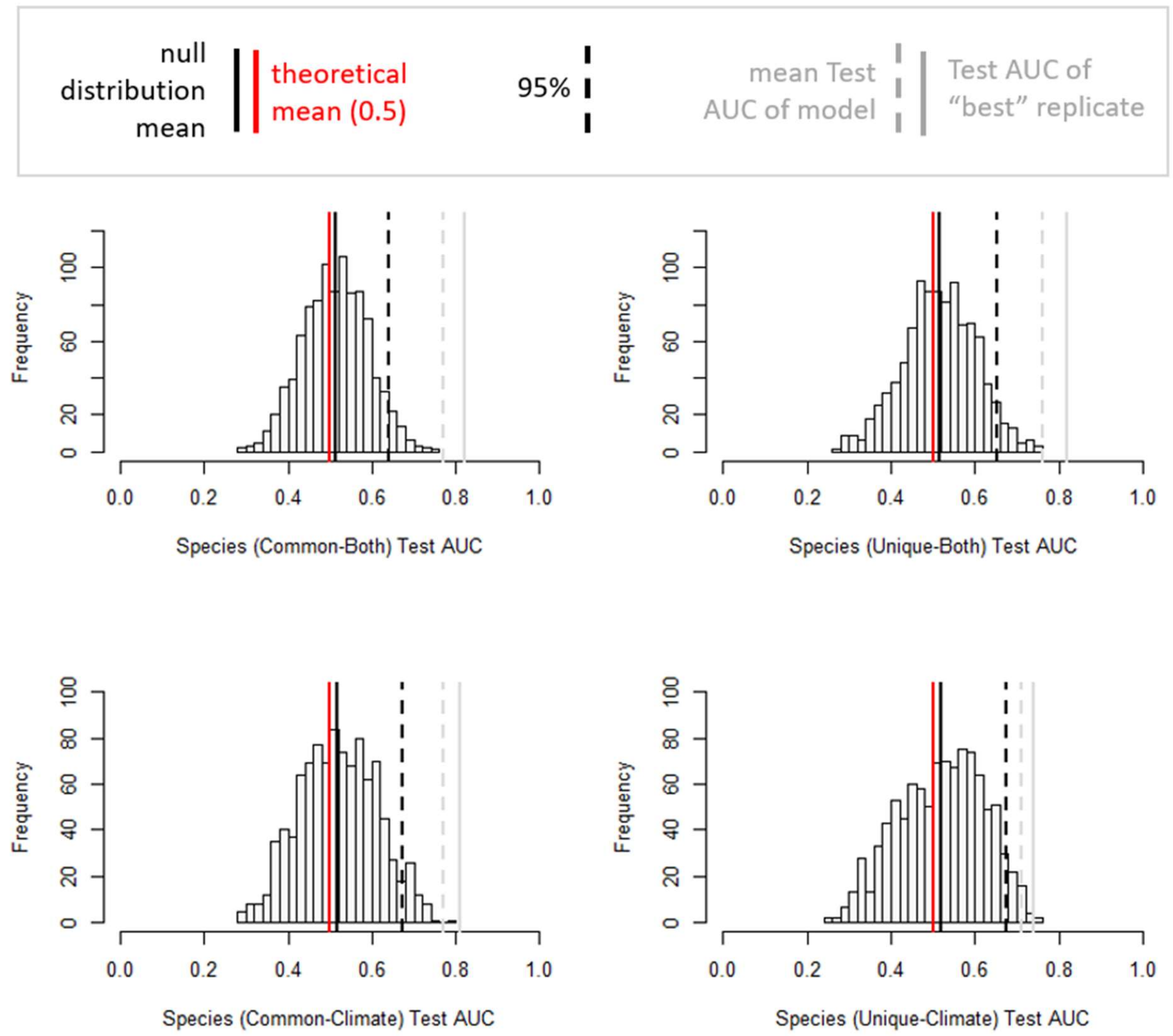

S8 Fig: Null Distribution of Test AUC values for southern models.

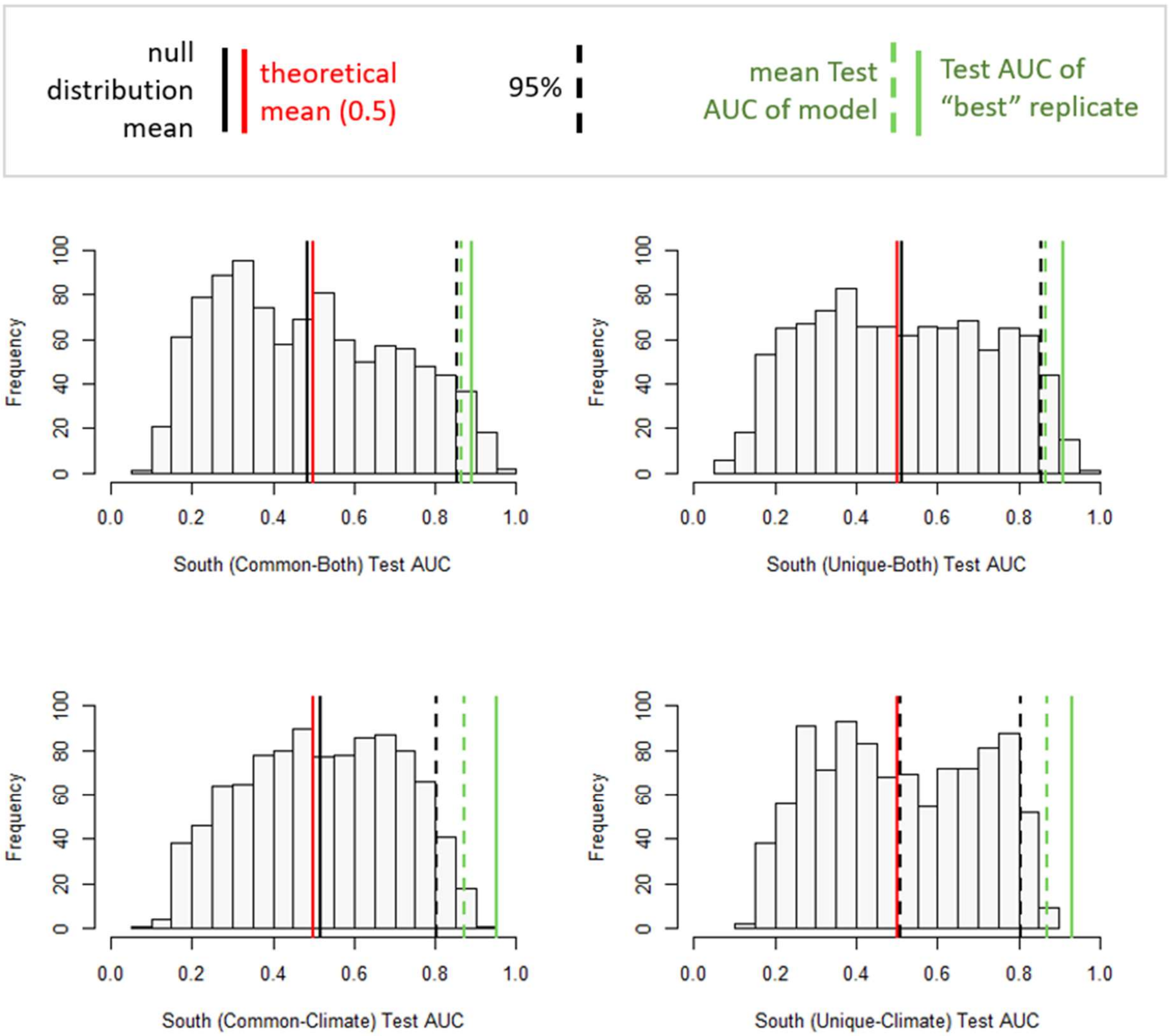

S9 Fig: Null Distribution of Test AUC values for central models.

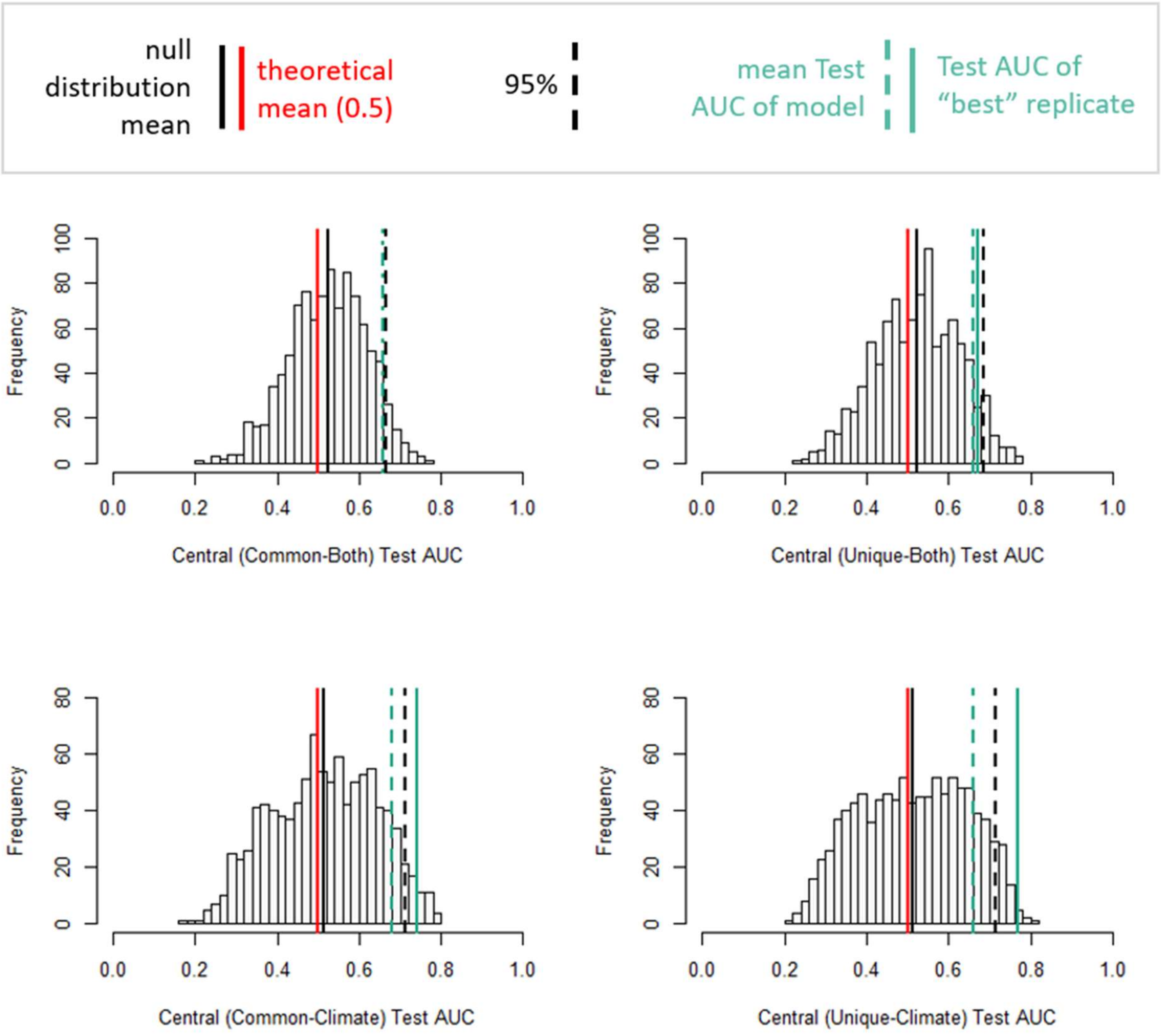

S10 Fig: Null Distribution of Test AUC values for northern models.

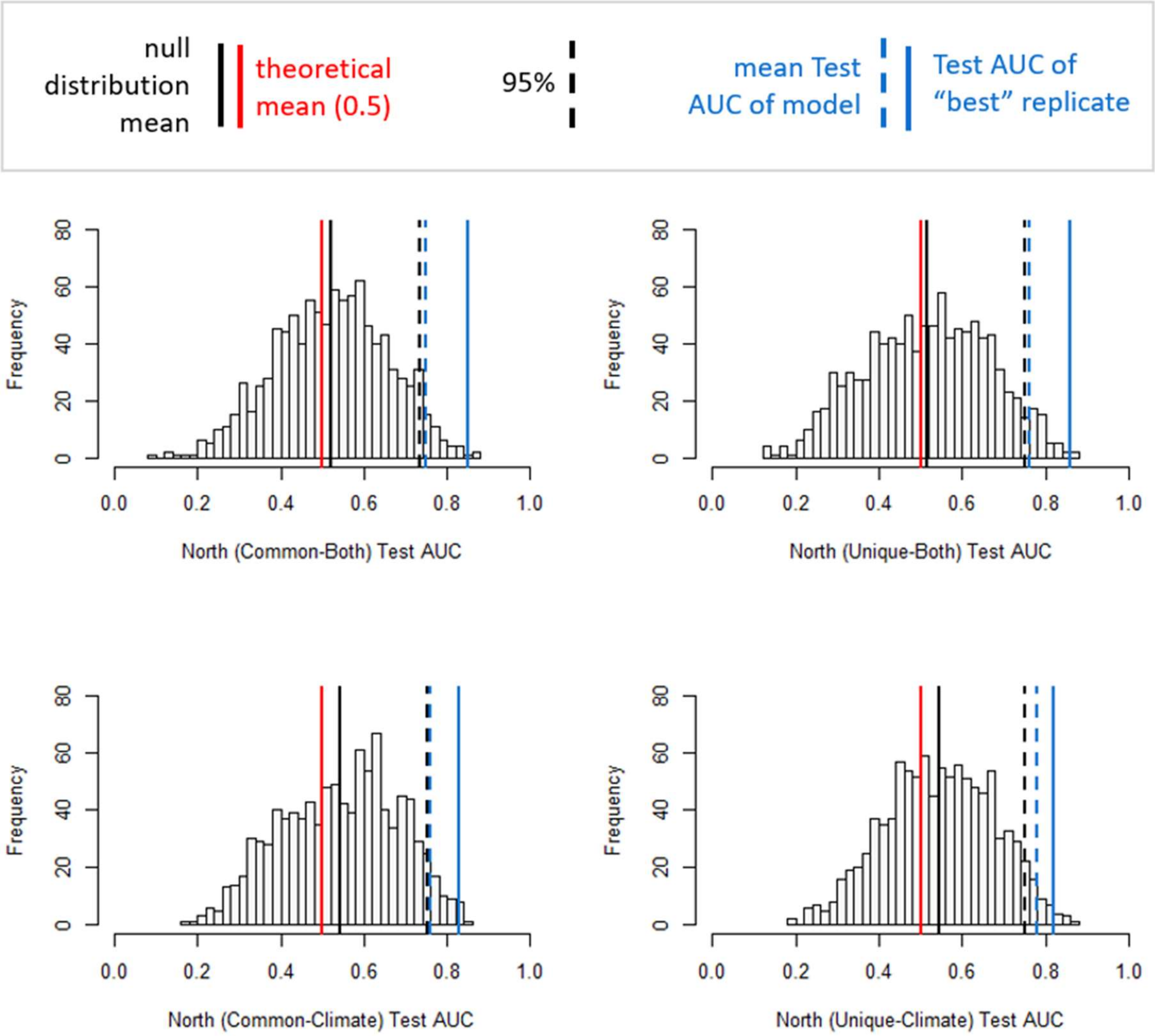

Supplement: S3 File — (PDF) [file pone.0274892.s003.pdf]
